# The therapeutic potential of mesenchymal stem cells for cardiovascular diseases

**DOI:** 10.1038/s41419-020-2542-9

**Published:** 2020-05-11

**Authors:** Yajun Guo, Yunsheng Yu, Shijun Hu, Yueqiu Chen, Zhenya Shen

**Affiliations:** 1 Institute for Cardiovascular Science, Soochow University, Suzhou 215006, China; 2 Department of Cardiovascular Surgery of The First Affiliated Hospital, Soochow University, Suzhou 215006, China; 3 State Key Laboratory of Radiation Medicine and Protection, Medical College, Soochow University, Suzhou 215123, China; 4 Collaborative Innovation Center of Hematology, Soochow University, Suzhou 215006, China

**Keywords:** Mesenchymal stem cells, Cardiovascular diseases

## Abstract

Mesenchymal stem cells (MSCs) are derived from a wide range of sources and easily isolated and cultured. MSCs have the capacity for in vitro amplification and self-renewal, low immunogenicity and immunomodulatory properties, and under certain conditions, MSCs can be differentiated into a variety of cells. In the cardiovascular system, MSCs can protect the myocardium by reducing the level of inflammation, promoting the differentiation of myocardial cells around infarct areas and angiogenesis, increasing apoptosis resistance, and inhibiting fibrosis, which are ideal qualities for cardiovascular repair. Preclinical studies have shown that MSCs can be transplanted and improve cardiac repair, but challenges, such as their low rate of migration to the ischemic myocardium, low tissue retention, and low survival rate after transplantation, remain. This article reviews the potential and methods of MSC transplantation in the treatment of cardiovascular diseases (CVDs) and the challenges of the clinical use of MSCs.

## Facts

- MSCs ameliorate cardiovascular diseases with immunoregulatory ability, antifibrotic effect, and neovascularization features.
- MSCs exert therapeutic function in cardiovascular diseases primarily through paracrine activities.
- MSCs exert immunoregulatory function via the innate immune system and/or the acquired immune system.

## Open questions

- What is the difference among MSCs derived from bone marrow, umbilical cord, and adipose tissue in trials of CVDs?
- In vivo identification of the influence of CVD microenvironment on the transplanted MSCs, and explore the potential manipulation to overcome the influence.
- The consensus on how to culture the MSCs used in clinical need to be designed.

## Introduction

Cardiovascular diseases (CVDs) have become a major contributor to the global disease burden due to their high morbidity and mortality rates^1^. CVDs are caused by infectious factors and noninfectious factors. Infectious CVDs range from rheumatic heart disease to tuberculous pericarditis and HIV-induced disease, while noninfectious CVDs include hypertension, myocardial infarction (MI), stroke, and peripheral artery disease. In addition, the incidence of CVD caused by noninfectious factors, especially ischemic CVDs such as MI, is likely to increasing in the coming decades^2^.

After MI, a common type of CVD, myocardial cells die and lose their function. Moreover, myocardial fibrosis of the infarcted heart causes ventricular remodeling, which results in further heart failure. Despite scientific progress and advancements in surgical techniques, drug and surgical treatments can only delay the progression of chronic heart disease but cannot save the function of infarcted myocardial cells^3^. MI leads to the migration of macrophages, monocytes, and neutrophils to the infarct area, where they produce an inflammatory response. Myocyte necrosis and a subsequent load increase trigger the initiation of signal transduction processes that regulate cardiac repair and then form fibrous scar tissue, which ultimately leads to heart failure^4^. Because drugs and surgery can only relieve the symptoms of heart failure but cannot save dead heart cells, clinicians began to look for a new way to treat heart failure after MI. Heart transplantation remains the only cure for heart failure patients, but donor organs are scarce, and the expense of the operation limits the development of this method. Inspiring, the use of stem cells emerged as a promising treatment for heart disease a decade ago^5^.

Stem cells include embryonic stem cells and adult stem cells. Embryonic stem cells, which are pluripotent stem cells, can be differentiated into embryonic structures^6^. Adult stem cells are multipotent stem cells with the capacity for self-renewal that can differentiate into different cell types^7,8^. In 1970, Friedenstein^9^ discovered a rare type of stromal cells in human bone marrow that are now known as mesenchymal stem cells (MSCs). MSCs are widespread in many tissues beyond bone marrow, including adipose tissue^10^, lung tissue^11^, the synovial membrane^12^, the endometrium^13^, and peripheral blood^14^. At present, the MSCs most commonly used in clinical studies are mainly derived from bone marrow, adipose tissue and cord blood^5^. Bone marrow and adipose tissue are the main sources of MSCs because of the difficulty in separating MSCs from umbilical cord blood^15^. However, MSCs from different sources exhibit differences in immunophenotype, differentiation potential, transcriptome, proteome, and immunomodulatory activity, producing their specific characteristics and features in their application. MSCs in the bone marrow, which account for ~0.001–0.01% of nucleated cells, preferentially adhere to plastic surfaces, so they can be easily isolated from hematopoietic stem cells and amplified in vitro^16^.

## Properties of MSCs

**Fig. 1:**
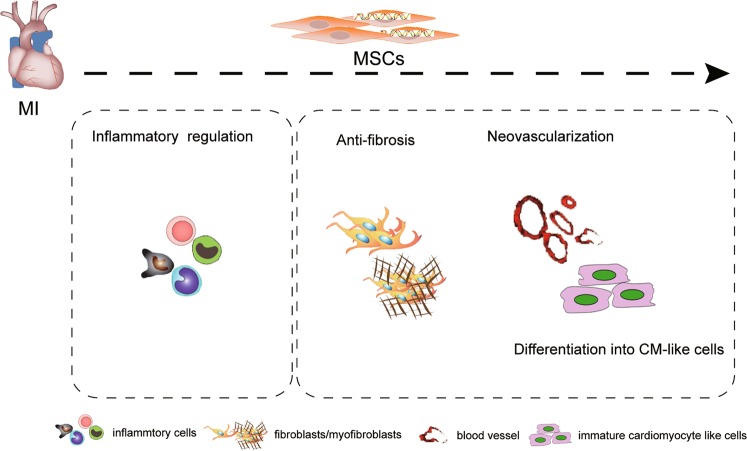
MSCs remedy cardiovascular disease through inflammatory regulation, anti-fibrosis, neovascularization and differentiation into cardiomyocyte-like cells. MI, myocardial infarction; CM, cardiomyocyte.

**Fig. 2:**
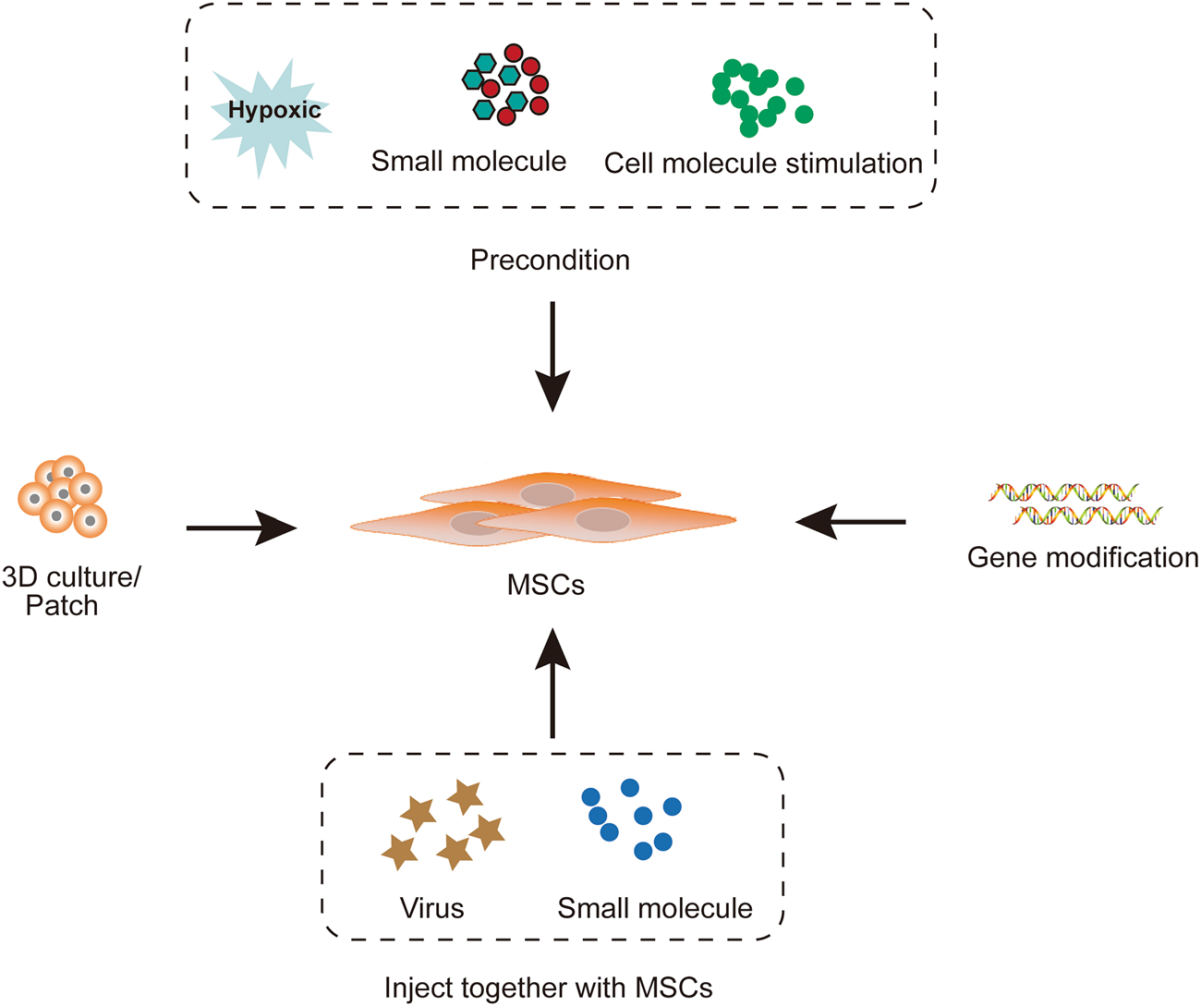
Strategies used to enhance the therapeutic effects of MSCs in cardiovascular diseases. 3D culture, patch including MSCs, precondition with hypoxic or molecules, gene modification, and injected together with virus overexpressing specific genes/shRNA or small molecules have been used to enhance therapeutic effects of MSCs.

MSCs play an important role in the therapy of CVD due to their special features, including their ability to differentiate into cardiovascular cells, immunomodulatory property, antifibrotic activity, and ability to undergo neovasculogenesis, which are summarized in Fig. 1. In addition to the differentiation of MSCs into cardiovascular progenitor cells, extracellular vesicles derived from MSCs are thought to mediate cellular functions; for example, MSC-derived extracellular vesicles were shown to enhance cardiomyocyte autophagy via the AMPK/mTOR and Akt/mTOR pathways^17^ and angiogenesis by the hypoxia inducible factor-1α (HIF-1α)/Jagged 1 pathway^18^, and reduce cell apoptosis and activate the cell survival signaling pathway through multiple microRNAs (miRs)^19–21^. Recently, various strategies to enhance the therapeutic effects of MSCs, including their genetic modification and combination with biological materials, have been developed^22–24^ (Fig. 2).

## Differentiation of MSCs into cardiomyocyte-like cells

After their transplantation, MSCs show a zonal distribution in myocardial tissue similar to that of cardiac myocytes. An increase in myocardial-specific marker proteins, such as troponin T, initially confirms the differentiation of MSCs into cardiomyocytes.

Human umbilical cord perivascular cells aggregate on cardiomyocyte feeder layers, generating contracting cell clusters within 1 week, and were the first type of MSC shown to do so^25^. Basic fibroblast growth factor (bFGF) can promote the migration and survival of bone marrow MSCs in vitro. Retrograde bFGF perfusion of the coronary vein can enhance the graft transplantation of MSCs, promote the phenotypic differentiation of MSCs to cardiomyocytes, restore cardiac function, and prevent adverse remodeling^26^. The cardiac differentiation of bone marrow MSCs can also be induced by the isolation and amplification of high-purity bone marrow MSCs with good growth dynamics into cardiomyocytes in vitro under combined stimulation with bFGF and hydrocortisone^27^.

In addition, MSCs has been genetically modified to induce their differentiation into cardiomyocyte-like cells. The treatment of MSCs with exogenous Jagged 1 activated the Notch1 signaling pathway and promoted multilineage differentiation^28^. The overexpression of miRNA1-2 in mouse MSCs induced by activation of the Wnt/β-catenin signaling pathway promoted the differentiation of MSCs into cardiomyocyte-like cells with enhanced Nkx2.5, cTnI, and GATA4 expression and reduced cytotoxicity^29^. Although the differentiation of MSCs into cardiomyocytes has been reported by researchers, it is widely accepted that the major effect of MSCs in the treatment of CVDs is dependent on paracrine effect, which will be present in the following paragraphs.

## Immunomodulatory properties of MSCs

**Fig. 3:**
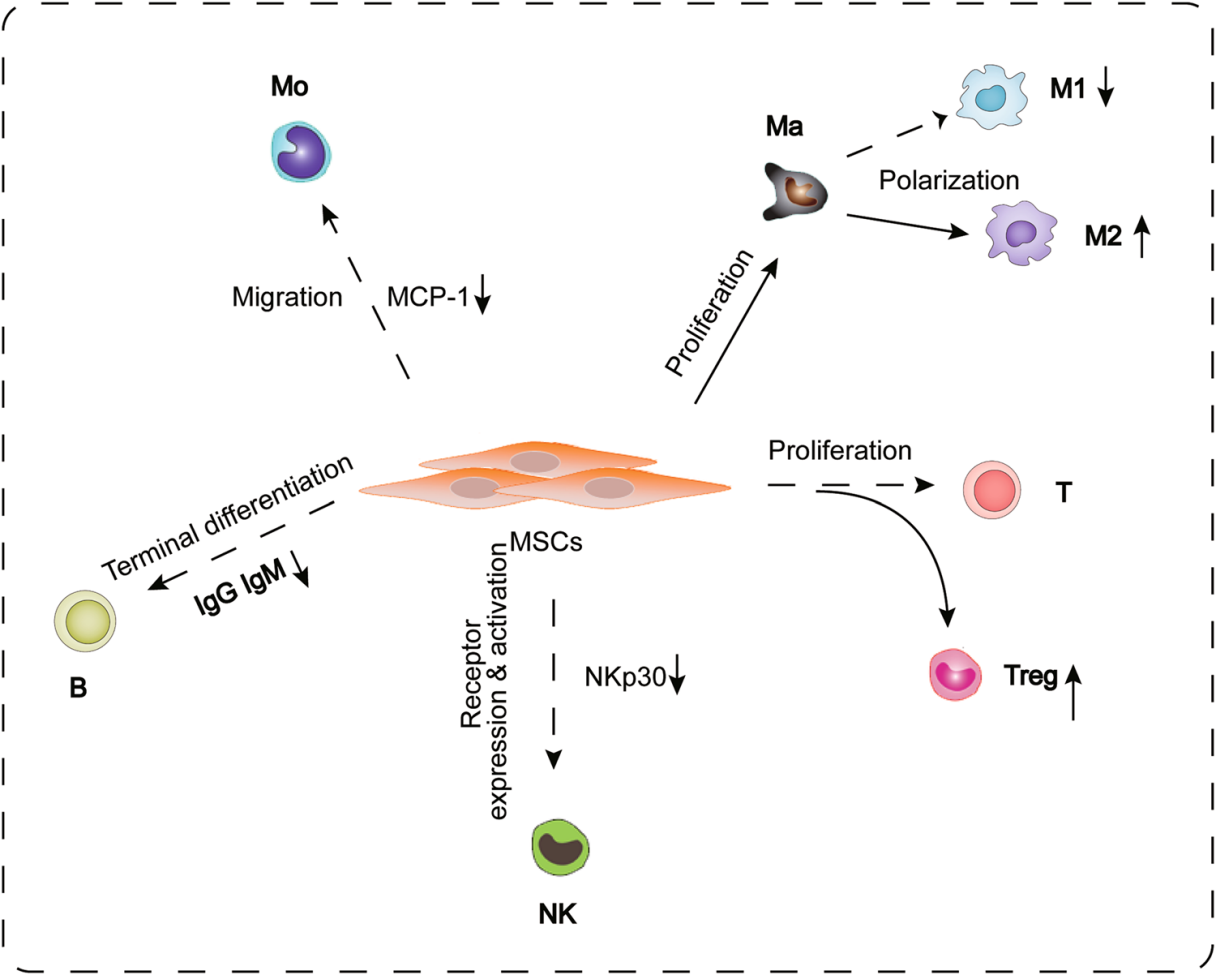
Cellular effect and ways of MSCs therapy in cardiovascular disease through inflammatory regelation. Mo, monocyte; Ma, macrophage; M1; subtype I of macrophage; M2; subtype II of macrophage; T, T cells; Treg, regulatory T cell; NK, natural killer cell; MCP-1, monocyte chemoattractant protein-1; IgG, immunoglobulin G; IgM, immunoglobulin M; NKp30, natural killer cell protein 30.

Previous studies have demonstrated that MSCs can regulate the inflammatory response by suppressing white blood cells and triggering anti-inflammatory subsets in innate immunity and adaptive immunity^30,31^ (Fig. 3). After MI, monocytes migrate to the infarct site, where they differentiate into macrophages, which secrete cytokines, chemokines, and growth factors to clear the infarct myocardial cells and apoptotic neutrophils. The activation of macrophages after MI produces different types of cells with different immune functions, mainly M1 macrophages, which produce interferon, tumor necrosis factor, and interleukin-23, and promote the inflammatory response and M2 macrophages, which are induced and activated by glucocorticoids or Th2-related cytokines, promoting cell proliferation, and angiogenesis^32^.

Miteva et al. demonstrated that the application of MSCs reduced the severity of myocarditis and decreased the number of proinflammatory monocytes expressing high or moderate levels of Ly6C; moreover, anti-inflammatory monocytes expressing low levels of Ly6C were increased in the blood, heart, and spleens of MSC-treated CVB3 mice^33^. Chiossone et al. demonstrated that MSCs could enhance the polarization of M2 macrophages through a prostaglandin E2-dependent mechanism and inhibit the proliferation of T cells^30^. The interaction between MSCs and macrophages promotes the high expression of CD206 and the anti-inflammatory cytokine IL-10 in macrophages, thereby inhibiting the inflammatory response in vitro, and macrophages cocultured with MSCs have higher phagocytotic activity^34^.

MSCs can inhibit T-cell proliferation in vitro and therefore can be used to treat severe graft-versus-host disease^35,36^. Studies have shown that when MSCs are cultured with T cells, indolamine-pyrrole 2-3-dioxygenase (IDO) is upregulated, leading to the consumption of tryptophan and the accumulation of its metabolites, thereby reducing the proliferation of T cells^37^. When natural killer (NK) cells were cultured in the presence of MSCs, activation of the receptors NKp30, NKp44, and NKG2D was suppressed^38^. Sotiropoulou et al. proved that MSCs inhibited the surface expression of 2B4 and CD132 without affecting the expression of CD16 in NK cells^39^.

Many other studies have shown that MSCs also participate in immune regulation through paracrine mechanisms^40^. Guo et al.^41^ found that after MSC transplantation, the expression of TNF-α, IL-1, and IL-6 and the apoptosis of myocardial cells were significantly reduced, leading to a significant improvement in cardiac function in MI mice. Ohnishi et al.^42^ discovered that the application of MSCs to a rat model of MI could reduce the levels of CD68-positive inflammatory cells and monocyte chemotactic protein-1 (MCP-1) in the myocardium, thereby improving cardiac function. In addition, conditioned medium derived from MSCs could reduce MCP-1-mediated damage to rat cardiac myocytes. Humoral factors produced by bone marrow stromal cells (BMSCs) inhibit the secretion of antigen-specific immunoglobulin M and immunoglobulin G1, thus inhibiting the terminal differentiation of B-cells^43^.

## Antifibrotic effect of MSCs

The most significant pathological feature after MI is myocardial fibrosis, which is characterized by excessive collagen deposition leading to stiffness with decreased diastolic and systolic function, resulting in myocardial scar formation^44,45^. Necrotic myocardial cells in the infarct area are replaced with fibroblasts, leading to ventricular remodeling, arrhythmias, or even death^46^.

MSCs can regulate matrix metalloproteinase to inhibit fibroblast activation, reduce extracellular matrix deposition, reduce left ventricular remodeling, and ultimately improve myocardial function. Previous studies have shown that HGF secreted by MSCs is an effective inhibitor of fibrosis and that HGF is the primary component responsible for the antifibrotic effect of MSCs in vitro^47^. MSCs also fight fibrosis through a paracrine mechanism. Studies have confirmed that MSCs transplanted into the area around MI released HGF through direct cell contact and inhibited miR-155-mediated profibrotic signaling, thus improving left ventricular remodeling and function in a mouse MI model^48^.

Moreover, several experiments have shown that gene modified MSCs have stronger antifibrotic effects. Silva et al. showed that insulin-like growth factor 1 (IGF-1)-overexpressing MSCs reduced the myofiber area in *Trypanosoma cruzi*-infected mice^49^. We previously demonstrated that miR133-overexpressing MSCs reduced fibrosis in MI through inhibiting Snail 1, a master regulator of epithelial-to-mesenchymal transition (EMT), and induced fibrogenesis during developmental and disease processes^50^. Furthermore, some studies showed that a hydrogel derived from decellularized bone extracellular matrix enhanced osteogenesis and cartilage regeneration in dental pulp stem cells or BMSCs^51,52^. Unlike bone or cartilage, the heart is a specialized organ that undergoes involuntary contraction, pumping blood to the rest of the body. Excessive collagen deposition interrupts myocyte–myocyte interactions in the myocardium and leads to stiffness with decreased diastolic and systolic function^53^.

## Neovascularization capacity of MSCs

Insufficient vessel growth associated with ischemia remains an unresolved issue in CVD, and the formation of new blood vessels is the basis of tissue repair^18^. Studies have shown that bone marrow-derived pluripotent stem cells play an important role in the formation of blood vessels by stimulating the formation of vascular networks by endothelial cells^54^.

The expression of MSC markers has been detected on the surfaces of native, noncultured perivascular cells. Thus, blood vessel walls harbor a reserve of progenitor cells that may be integral to the origin of elusive MSCs and other related adult stem cells^55^. Preclinical in vivo studies have shown that some transplanted BMSCs lose their smooth muscle phenotype and differentiate into endothelial cells, increasing the microvascular density and improving cardiac function in rat models of MI^56^. However, some research groups believe that transplanted BMSCs promote cardiac repair and angiogenesis in models of cardiac injury mainly through indirect paracrine signaling primarily through the release of angiogenesis factors and arteriogenesis factors^57^. Tang et al.^58^ compared the recovery effects of transplantation with simple BMSCs, treatment with pure VEGF, and combined VEGF treatment and BMSC transplantation in rats after MI, the results of which showed that joint VEGF treatment and BMSC transplantation significantly increased vascular density (80%) and reduced the collagen content (33%). The transplanted cells promoted vascular repair and the strongest inhibitory effect on cardiac function decline.

Other studies have shown that MSCs participate in enhancing angiogenesis through paracrine mechanisms. Ju et al. showed that cardiac MSC-secreted exosomes improved cardiac function by enhancing capillary density and cardiomyocyte proliferation^20^. In addition, Gonzalez-King et al. demonstrated that exosomes derived from MSCs, especially MSCs under hypoxic conditions, induced angiogenesis in an in vitro model of capillary-like tube formation, and a Matrigel plug assay showed that these exosomes induced angiogenesis in vivo through HIF-1α^18^. In addition to genes that are overexpressed in MSCs, Gao et al. added a new self-assembling peptide—the angiogenic polypeptide SVVYGLR—to the carboxyl terminal of RADA16 to evaluate the angiogenic effect of MSCs^59^.

## Clinical trials

On the basis of experiments in model and the preclinical trials mentioned above, MSC transplantation therapy is widely used for the treatment of CVDs, especially MI. Many completed and ongoing clinical trials have been conducted to investigate the efficacy of MSCs derived from many kinds of tissue (Table 1).

**Table 1:** Completed and ongoing trials of MSCs in heart disease.

Clinicaltrials.gov identifier	Disease type	Study design	Route of delivery	Endpoint	Enrolled number	Type of MSCs	Status
NCT00114452^60^	Acute MI	Randomized; parallel assigned; phase I	Intravenous injection	Adverse event rates	53	Adult hMSCs	Completed
NCT03902067	Acute MI	Randomized; parallel assigned; phase I	Injection into blood vessels through an administration catheter	Safety, LVEF	40	UC-MSCs	Not yet recruiting
NCT03533153	MI	Randomized; parallel assigned; phase I/ II	Intravenous injection	Heart function; infarct size	90	Clinical-grade WJ-MSCs	Not yet recruiting
NCT02323477^61^	Chronic ischemic cardiomyopathy	Randomized; parallel assigned; single; phase I/ II	Intramyocardial injection	LVEF; LVED; LVES	79	UC-MSCs	Unknown
NCT02503280	Chronic ischemic left ventricular dysfunction	Randomized; parallel assigned; single; phase I/ II	Transendocardial injection	Safety; cardiac function	55	hMSCs	Not yet recruiting
NCT01652209	Acute MI	Randomized; parallel assigned; open label; phase III	Intramyocardial injection	Efficacy and safety	135	BM-MSCs	Recruiting
NCT02013674	Chronic ischemic left ventricular dysfunction	Randomized; parallel assigned; phase II	Injected through BioCardia helical infusion system	Safety; efficacy assessment	30	Allogeneic hMSCs	Unknown
NCT02467387^85^	Nonischemic heart failure	Single-blind, randomized; phase II	Intravenously injection	6-minute walking distance; LVEF	22	BM- MSCs	Completed
NCT01739777^62^	Chronic stable heart failure	Randomized controlled trial; phase I/ II	Intracoronary injection	Safety, NYHA; LVEF	22	UC-MSCs	Completed
NCT01291329^86^	ST-elevation MI	Randomized; parallel assigned; phase II	Intracoronary injection	Safety; LVEF	160	Umbilical WJ-MSCs	Completed
NCT00883727	ST-elevation acute MI	Randomized; parallel assigned; triple; phase I/ II	Intravenous injection	Regional myocardial perfusion and infarct size	20	Adult allogenic MSCs	Completed
NCT01392105^87^	Acute MI	Randomized; parallel assigned; open label; phase II/ III	Intracoronary injection	LVEF	80	BM-hMSCs	Completed

*MI* myocardial infarction, *NYHA* New York Heart Association functional class, *LVEF* left ventricular ejection fraction, *LVED* left ventricular end diastolic diameter, *LVES* left ventricular systolic diameter.

Adult allogenic MSCs derived from bone marrow are the most widely used cells in the treatment of CVD. A United States clinical trial conducted by Joshua Hare and colleagues in 2005 (NCT00114452) was the first clinical trial to use MSCs in MI^60^. This study was a randomized and parallel assigned clinical trial of the use of MSCs for the treatment of acute MI (heart attack). The trial provided pivotal safety and provisional efficacy data for the use of allogeneic bone marrow-derived stem cells in postinfarction patients. Another clinical trial reported by Ankara University in 2015 was a phase I/II, controlled, multicenter, randomized clinical study of the intramyocardial delivery of allogeneic human umbilical cord mesenchymal stem cells (HUC-MSCs) in 79 patients with chronic ischemic cardiomyopathy^61^ that aimed to investigate the efficacy and safety of HUC-MSCs. Another study (NCT01739777) was conducted by Jorge Bartolucci and colleagues to evaluate the safety and efficacy of the intravenous infusion of UC-MSCs in patients with chronic stable heart failure. The study, a phase I, randomized, double-blind clinical trial, showed that the intravenous infusion of UC-MSCs was safe and improved left ventricular function, functional status, and quality of life^62^.

The above studies indicate that MSC treatment is safe and can improve cardiac perfusion after MI. More importantly, the clinical trials in Table 1 show the favorable safety profile of MSC transplantation and that no tumor development was reported with MSC transplantation. However, the systematic application of MSCs carries some potential risks, such as embolism and inflammation. Although many clinical trials have been conducted by researchers in CVDs, however, this kind of trial is still in a very early stage. The main purpose and basic content are to investigate the safety and efficacy of the transplanted MSCs, a clear comparison and definition of results have not been reported to the best of our knowledge. Perhaps trials are needed in the future to determine the difference between bone marrow MSCs, adipose-derived MSCs and umbilical cord-derived MSCs.

## Problems and prospects

Much research has proven the feasibility, safety, and efficiency of MSC-based therapy for CVD. MSCs offer new hope for the treatment of CVDs, especially MI, but some problems remain to be solved. The complexity and versatility in the sources of MSCs, and there is no consensus on how to culture the MSCs, resulting in a different therapeutic effect in CVDs. MSCs derived from bone marrow, adipose tissue, and umbilical cord have different differentiation ability and immunoregulatory effects in CVDs^63,64^. The difference in culture conditions may influence the properties of MSCs, such as activity and immunoregulatory ability. However, MSCs must meet the basic features, such as cell phenotype and the differentiation ability toward adipogenic, osteogenic and chondrogenic lineages. A portion of the therapeutic effect of MSCs depends on their transplantation and ability to survive in the graft site. However, the stem cell retention rate is low, and the immunogenicity of transplanted cells is disturbed by the hostile ischemic environment, so cardiac function is only slightly improved^65,66^.

As some studies have shown that MSCs have a low survival rate in the cardiac environment and that a large proportion of transplanted cells may disappear soon after transplantation, further improvements to enhance the survival rate of MSCs are needed^67^. Chu et al. constructed a novel collagen-nanomaterial-drug hybrid scaffold based on GO-PEG-mediated quercetin-modified acellular dermal matrix (ADM) with a cell-adhesive surface to accelerate MSC attachment and proliferation^68^. In addition, we constructed a silk fibroin microsphere-based injectable alginate hydrogel that could spatially and temporally control the release of IGF-1 in a rat model of MI^69^. Therefore, the combined application of nanoparticles or sustained releasable hydrogels with MSCs may serve as a promising option for the treatment of ischemic cardiomyopathy on the basis of cell therapy.

Extensive in vitro expansion is a prerequisite to obtain the number of cells required for cell therapy. However, spontaneous senescence, a process that limits cell death, may be a key issue in the long-term in vitro expansion of MSCs^70^. Recombinant collagen I peptide was added to culture medium to enhance the proliferation of MSCs through enhancing the expression of genes encoding proteins associated with cell adhesion^71^. EGF induces MSC proliferation through the EGFR/ERK and AKT pathways, and the G1/S transition was shown to be stimulated by the upregulation of cyclin D1 expression and inhibition of p16 expression^72^. Although MSCs appear to be genetically stable, they are prone to chromosomal abnormalities and malignant transformation under long-term culture in vitro^73^. Chromosomal aberrations have also been detected in pluripotent stem cells^74^. Therefore, in addition to the proliferation of MSCs, the reasonable and actively controlled proliferation of MSCs is equally important.

MSCs derived from different tissues have been used clinically, and many approaches have been done to enhance the therapeutic effect in preclinical studies. Little is known about the unfavorable effects of MSCs in the heart. It was reported by Shani et al. that the environment of the infarcted myocardium drove MSCs toward a proinflammatory phenotype and reduced the survival and paracrine effect of MSCs via TLR4^75^. Meanwhile, other reports to improve the complex microenvironment in heart diseases have been published. Myocardial transfection of HIF-1α via an adenoviral vector or injection of p38MAPK inhibitor (SB203580) was used to optimize to effects of transplanted MSCs^76,77^. The impact of heart disease environment on the transplanted MSCs and the approach to improve to transplantation-site microenvironment are worth exploring in the future.

Although some studies have reported that MSCs have antifibrotic and immunoregulatory effects after MI, few clinical studies of the treatment of MI with MSC transplantation have been conducted^22,30^. A large number of clinical trials should be conducted to determine the optimal MSC dose and delivery route to improve the survival rate of patients with MI and lay the foundation for solving other problems^78,79^. In addition to their effect in MI, MSCs and MSC-derived extracellular vesicles have important effects on other CVDs, such as aortic aneurysm and atherosclerosis^80–84^.

## Conclusions

MSCs have the advantages of a wide range of sources, easy isolation and amplification, and low immunogenicity. In addition, transplanted MSCs can migrate to infarcted myocardial tissue, reduce the inflammatory response, reduce fibrosis, promote the formation of new blood vessels, and differentiate into cardiomyocyte-like cells, ultimately contributing to the repair of infarcted myocardium. Despite their advantages for the treatment of CVDs, the use of MSCs still faces some challenges, such as the poor targeted migration and low survival rate of MSCs in the ischemic myocardium. The feasibility and safety of MSC therapy have been tested in clinical trials, but the optimal MSC dose and delivery route for the treatment of MI should be studied. Although some problems remain for the use of MSCs to treat CVDs, MSCs are still a promising form of cell therapy.
